# Sex-Specific Risk Factors and Clinical Dementia Outcomes for White Matter Hyperintensities in a large South Korean Cohort

**DOI:** 10.21203/rs.3.rs-4473148/v1

**Published:** 2024-06-12

**Authors:** Noah Schweitzer, Sang Joon Son, Rebecca C. Thurston, Jinghang Li, Chang-Le Chen, Howard Aizenstein, Shaolin Yang, Bistra Iordanova, Chang Hyung Hong, Hyun Woong Roh, Yong Hyuk Cho, Sunhwa Hong, You Jin Nam, Dong Yun Lee, Bumhee Park, Na-Rae Kim, Jin Wook Choi, Jaeyoun Cheong, Sang Woon Seo, Young-Sil An, So Young Moon, Seung Jin Han, Minjie Wu

**Affiliations:** University of Pittsburgh; Ajou University School of Medicine; University of Pittsburgh School of Medicine; University of Pittsburgh; University of Pittsburgh; University of Pittsburgh; University of Pittsburgh; University of Pittsburgh; Ajou University School of Medicine; Ajou University School of Medicine; Ajou University School of Medicine; Ajou University School of Medicine; Ajou University School of Medicine; Ajou University School of Medicine; Ajou University School of Medicine; Ajou University School of Medicine; Ajou University School of Medicine; Ajou University School of Medicine; Samsung Medical Centre, Sungkyunkwan University School of Medicine; Ajou University School of Medicine; Ajou University School of Medicine; Ajou University School of Medicine; University of Pittsburgh School of Medicine

## Abstract

**Objective:**

White matter hyperintensities (WMH) on brain MRI images are the most common feature of cerebral small vessel disease (CSVD). Studies have yielded divergent findings on the modifiable risk factors for WMH and WMH’s impact on cognitive decline. Mounting evidence suggests sex differences in WMH burden and subsequent effects on cognition. Thus, we aimed to identify sex-specific modifiable risk factors for WMH. We then explored whether there were sex-specific associations of WMH to longitudinal clinical dementia outcomes.

**Methods:**

Participants aged 49–89 years were recruited at memory clinics and underwent a T2-weighted fluid-attenuated inversion recovery (FLAIR) 3T MRI scan to measure WMH volume. Participants were then recruited for two additional follow-up visits, 1–2 years apart, where clinical dementia rating sum of boxes (CDR-SB) scores were measured. We first explored which known modifiable risk factors for WMH were significant when tested for a sex-interaction effect. We additionally tested which risk factors were significant when stratified by sex. We then tested to see whether WMH is longitudinally associated with clinical dementia that is sex-specific.

**Results:**

The study utilized data from 713 participants (241 males, 472 females) with a mean age of 72.3 years and 72.8 years for males and females, respectively. 57.3% and 59.5% of participants were diagnosed with mild cognitive impairment (MCI) for males and females, respectively. 40.7% and 39.4% were diagnosed with dementia for males and females, respectively. Of the 713 participants, 181 participants had CDR-SB scores available for three longitudinal time points. Compared to males, females showed stronger association of age to WMH volume. Type 2 Diabetes was associated with greater WMH burden in females but not males. Finally, baseline WMH burden was associated with worse clinical dementia outcomes longitudinally in females but not in males.

**Discussion:**

Elderly females have an accelerated increase in cerebrovascular burden as they age, and subsequently are more vulnerable to clinical dementia decline due to CSVD. Additionally, females are more susceptible to the cerebrovascular consequences of diabetes. These findings emphasize the importance of considering sex when examining the consequences of CSVD. Future research should explore the underlying mechanisms driving these sex differences and personalized prevention and treatment strategies.

**Clinical trial registration:**

The BICWALZS is registered in the Korean National Clinical Trial Registry (Clinical Research Information Service; identifier, KCT0003391). Registration Date 2018/12/14.

## Introduction

Cerebral small vessel disease (CSVD) is a common cause of stroke and cognitive impairment in older adults. White matter hyperintensities (WMH) observed on brain T2-weighted fluid-attenuated inversion recovery (T2-FLAIR) magnetic resonance imaging (MRI) are surrogate markers of CSVD. While there is clear evidence that WMH leads to cognitive decline, the magnitude of its relationship to cognition and to the rate of cognitive decline varies considerably across individuals^1^. Moreover, studies have yielded divergent findings on the risk factors for development and progression of WMH^2^. Sex appears to be an important moderator in how risk factors are related to WMH incidence and severity, yet few have reported sex-specific risk factor differences^3^. A comprehensive understanding of sex-specific modifiable risk factors for WMH can inform improved diagnostics and targeted treatment.

Studies reporting sex-specific differences for the consequences of WMH have been inconsistent. A majority of studies found postmenopausal, elder females to have higher WMHV burden compared to males^4–10^, yet some have observed no differences^11^ or that males have higher WMH burden^12, 13^. Previous studies have also reported that modifiable risk factors for WMH are sex-specific. Hypertension and higher body mass index (BMI) have been observed to have a stronger association with WMH burden in males compared to females^14–17^. Studies have reported that diabetes and smoking are risk factors for females but not males^8, 18–20^. It has also been reported that WMH is associated with worse cognitive and clinical outcomes in females compared to males^16^.

Thus, it is necessary to identify modifiable risk factors of CSVD have sex-specific associations with CSVD, which in turn can inform approaches to the prevention and treatment of dementia. In this study of 713 predominantly cognitively impaired participants, we tested whether associations of modifiable risk factors for WMH varied by sex. We then tested any sex differences in longitudinal associations of WMH to clinical dementia outcomes. We hypothesized that females would have a stronger association of age with WMH, the modifiable risk factors would be sex-specific, and that WMH would have a stronger effect on clinical dementia outcomes in females.

## Methods

### Participants

This study was a part of the ongoing Biobank Innovations for Chronic Cerebrovascular Disease With ALZheimer’s Disease Study (BICWALZS) and the Centre for Convergence Research of Neurological Disorders. The BICWALZS was planned and initiated in October 2016 by the Korea Disease Control and Prevention Agency for the Korea Biobank Project, a national innovative biobanking program that fosters biomedical and healthcare research and development infrastructure. The original goal was to facilitate, regulate, and ensure the optimal use of human biological specimens for research from real-world data in the fields of subjective cognitive decline (SCD), mild cognitive impairment (MCI), Alzheimer’s disease (AD), and subcortical vascular dementia (SVaD).

All participants underwent Clinical Dementia Rating (CDR) global score and sum of boxes of CDR (CDR-SB). The CDR is obtained by interviewing patients and their care givers and captures cognition and function. It assesses six domains (memory, orientation, judgment and problem solving, community affairs, home and hobbies, and personal care) and the score for each domain range from 0 to 3, with a higher score indicating greater impairment. Then, CDR-SB score range from 0 to 18 and is a validated outcome measure used in clinical trials of dementia^21, 22^.

The clinical diagnosis criteria used for this study were as follows: SCD criteria included self-and/or informant reports of cognitive decline but no objective impairment in cognitive tasks (no less than − 1.5 SD in each of the neurocognitive test domains and CDR = 0)^23^; patients with MCI were evaluated based on a CDR^21^ score of 0.5, the expanded Mayo Clinic criteria^24^, patients with AD dementia were evaluated using the National Institute on Aging-Alzheimer’s Association Core Clinical Probable AD Dementia Criteria^25^; and subcortical vascular dementia (SVaD) was evaluated based on above-moderate WMH and vascular dementia criteria in accordance with the Diagnostic Statistical Manual of Mental Disorders, fifth edition^26^. Patients with a history of neurological or medical conditions such as territorial cerebral infarction, intracranial hemorrhage, Parkinson’s disease, heart failure, renal failure, or others that could interfere with the study were excluded. The presence or absence of diabetes, hypertension, and hyperlipidemia was based on the clinical history of treatment with the diagnosis by a physician. Blood pressure, pulse pressure, body mass index and smoking status also were evaluated.

The BICWALZS is registered in the Korean National Clinical Trial Registry (Clinical Research Information Service; identifier, KCT0003391). The study was approved by the Institutional Review Board of Ajou University Hospital (AJOUIRB-SUR-2021–038), and written informed consent was obtained from all the participants and caregivers. Participants from the BICWALZS were recruited at the memory clinics of Ajou University Hospital and Suwon Community Geriatric Centers in South Korea. All the participants were Korean (Eastern Asian ethnicity). Among these individuals, we used data from 713 participants with brain MRI, amyloid PET, APOE, CDR, and blood laboratory assessments. Within this cohort, 181 participants had two additional follow-up visits where their CDR was measured.

### Blood sampling and laboratory assessments

Blood samples were collected by venipuncture after an overnight fast in the morning. Blood laboratory tests included HbA_1c_, serum lipid, homocysteine, and thyroid function tests.

### APOE genotyping

Informed consent was obtained from all participants regarding the collection and genotyping of blood genomic DNA. Genomic DNA was isolated from the blood samples, and single-nucleotide polymorphism (SNP) genotyping was performed by DNA Link, Inc. (Seoul, Korea) using the Affymetrix Axiom KORV1.0–96 Array (Thermo Fisher Scientific, Waltham, MA, USA) according to the manufacturer’s protocol. The APOE genotypes were derived from rs429358 and rs7412, which were included in the array.

### Amyloid PET acquisition and measurement of amyloid deposition

^18^ F-flutemetamol PET scan was performed on a Discovery STE/690 PET/CT scanner (GE, Milwaukee, WI, USA), with the same protocol used on all participants. ^18^F-flutemetamol was injected into the antecubital vein as a bolus (mean dose, 185 MBq). After 90 min, a 20-min PET scan (4 × 5 min dynamic frames) was performed. The PET sequence parameters are listed in Supplementary Table 1. ^18^F-flutemetamol PET scans were co-registered to individual MRI scans, which were normalized to a T1-weighted MRI template using transformation parameters. To quantify ^18^F-flutemetamol retention, the standard uptake value ratio (SUVR) was obtained using the pons as a reference region. Global cortical ^18^F-flutemetamol retention was calculated using an automated anatomical labeling (AAL) atlas.

### Image acquisition and MR data processing for white matter hyperintensities

Participants completed the baseline MRI scans on a GE Discovery MR750w 3T scanner or a Philips Achieva 3T Scanner, including the following two sequences: a three-dimensional (3D) magnetization-prepared rapid gradient echo (MPRAGE) T1-weighted sequence and a T2-weighted (T2w) fluid-attenuated inversion recovery (FLAIR) sequence. The MRI sequence parameters are listed in Supplementary Table 1. To quantify the WMH on T2w FLAIR images, we leveraged a pretrained deep learning model described in our previous study^27^. Brie y, the deep learning segmentation model consists of a transformer-based encoder and a convolutional decoder to ensure a larger receptive field for lesion segmentation. The model was trained on an unparalleled dataset including FLAIR images acquired at 1.5T, 3T and 7T with significant data augmentation incorporating commonly seen MR artifacts, such as, noise, inhomogeneity, and minor ghosting. FreeSurfer (version 7.1.1, https://surfer.nmr.mgh.harvard.edu/) was used to calculate intracranial volume (ICV). The total WMH volume (WMHV) was normalized by the ICV [WMHV = WMH/ICV] and log-transformed for analysis.

### Statistical analysis

We performed two analyses using multivariate linear regression: a sex-interaction analysis and a main effect analysis. Sex-specific risk factors for WMH were tested using multivariate linear regression models performed in R (version 4.3.1 https://www.R-project.org). Predictors of interest included age, diabetes status, hypertension status, body mass index (BMI), cardiovascular risks (pulse pressure, systolic and diastolic blood pressure, low-density and high-density lipid levels), thyroid stimulation hormone (TSH) levels, and amyloid β (Aβ) burden (global ^18^F-flutemetamol SUVR). TSH, HbA_1c_ and homocysteine were log-transformed due to rightward skew. We first performed a sex-interaction analysis to test for individual risk factor’s interaction with sex on WMHV. Risk factors tested included age, hypertension status, diabetes status, HbA_1c_, pulse pressure, diastolic and systolic blood pressure, homocysteine, TSH, APOE4 status, HDL, and LDL. In the main effect analysis, we tested individual risk factors stratified by sex. The analyses controlled for scanner site and cognitive diagnosis (i.e., no cognitive decline, SCD/MCI, dementia). Multiple comparisons correction were performed for each analysis using the Benjamini-Hochberg false discovery rate method (FDR)^28^.

Of all the 713 participants with cross-sectional data available, there were 181 participants who completed two additional follow-up visits and obtained additional CDR sum of boxes (CDR-SB) scores. Follow-up visits were acquired approximately one year after the prior visit. To analyze the association between WMHV and clinical outcomes longitudinally, we utilized a Linear Mixed Effects model (LME) to test for associations of CDR-SB with the interaction effect of WMHV*visit time (baseline, follow-up, visit 3), Aβ burden*visit time, stratified by sex. The LME model controlled for fixed effects of age and scanner site, as well as a random slope for each participant. LME models were implemented with the lmer function from the lme4 package in R^29^. To visualize results, we used the function ‘plot_model’ in the R package sjPlot to generate plots of the marginal effects of WMHV on visit time^30^.

## Results

Table 1 displays the characteristics of the entire cohort stratified by sex. Participants were 241 males and 472 females, with an average age 72.3 years and 72.8 years for males and females, respectively. 57.3% and 59.5% of participants were diagnosed with SCD or MCI for males and females, respectively. 40.7% and 39.4% were diagnosed with dementia for males and females, respectively. There were no significant sex differences in the proportion of those with SCD/MCI vs. dementia vs. no cognitive decline at baseline. Males had a significantly higher number of years of education, proportion with diabetes, and LDL and HDL levels. Females had significantly higher homocysteine levels. Supplementary Table 2 displays the characteristics of the participants in the cross-sectional cohort who had two additional follow-up visits with a Clinical Dementia Rating Sum of Boxes (CDR-SB) measured versus those who did not have longitudinal measures. No demographic characteristics varied significantly between the two subgroups. Additionally, CDR-SB scores did not significantly differ between male and female at any of the three longitudinal time points (not shown, *p* = 0.97, 0.66, and 0.91 for visit at baseline, visit 2, and visit 3, respectively).

**Figure 1:**
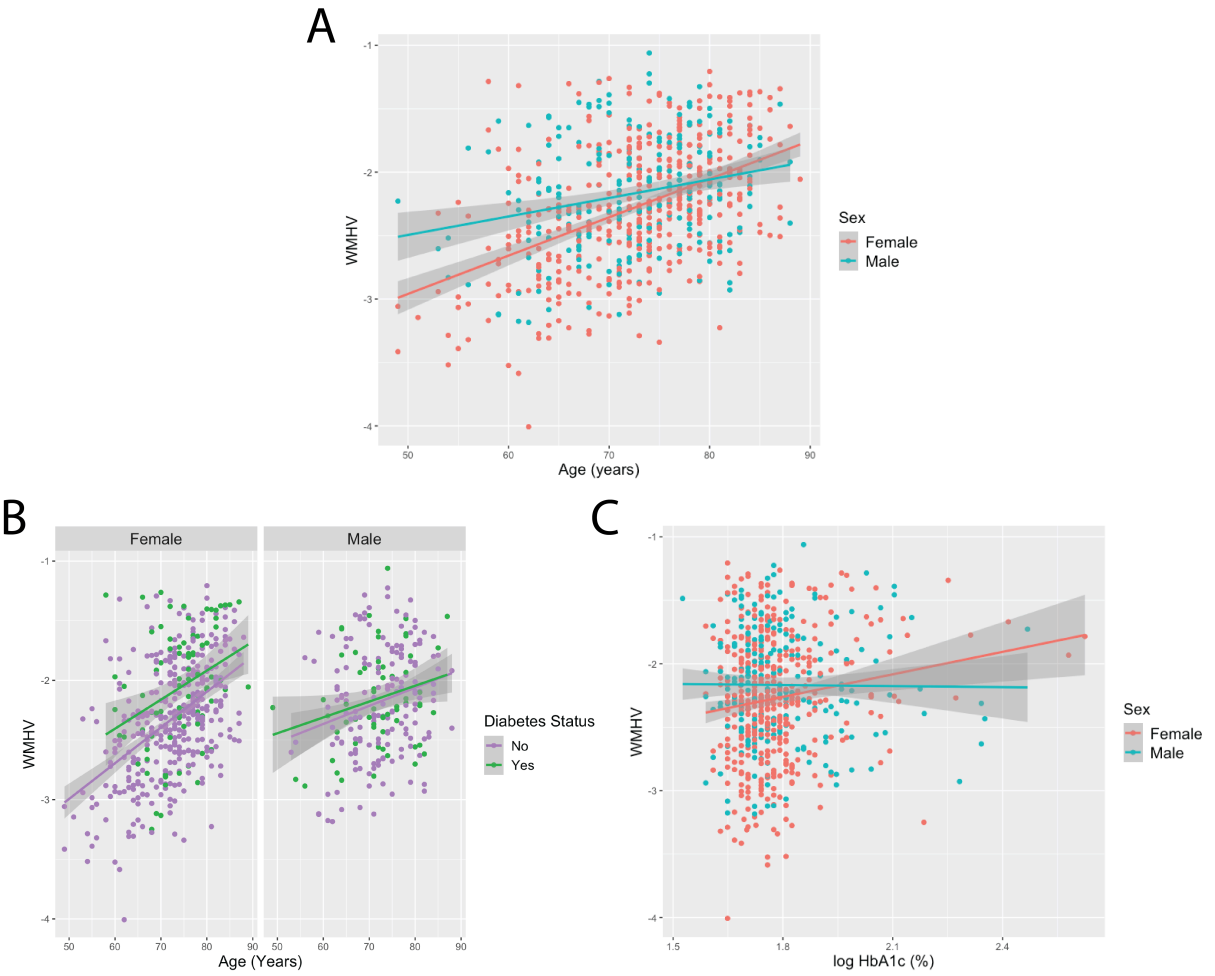
Elderly females appear to have an accelerated increase in cerebrovascular burden as they age and are more susceptible to the cerebrovascular consequences of diabetes. A.) Females are observed to have a higher increase in WMHV as they age compared to males. B.) Diabetic females were associated with larger WMHV compared to non-diabetic females. There were no differences observed for males. For visualization purposes, WMHV is plotted versus age and grouped by sex and Diabetes status. C.) Higher HbA_1c_ levels were associated with larger WMHV for females but not males, but the interaction effect for HbA_1c_*sex did not survive multiple comparisons. WMHV= white matter hyperintensity volume

We tested each WMH risk factor for a sex interaction effect controlling for scanner site and cognitive diagnosis. Two interaction effects tested survived multiple comparisons: age*sex and diabetes*sex (Table 2). WMHV increased at a higher rate as females aged compared to males (Fig. 1A). Diabetic females had higher WMHV compared to non-diabetic females whereas there were no group differences for males (Fig. 1B). Notably, HbA_1c_*sex was close to surviving multiple comparison (FDR corrected *p*-value = 0.07). Higher HbA_1c_ was associated with greater WMHV for females but not males (Fig. 1C).

**Figure 2:**
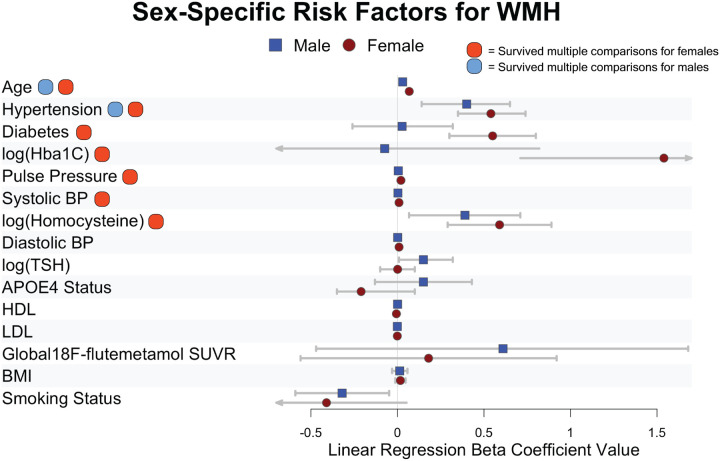
Sex-specific risk factors for WMH. Beta coefficient values from linear regression models for individual risk factors are displayed.

To visualize sex-specific risk architectures, we tested which risk factors were significantly associated with WMHV when stratified by sex and controlling for scanner site and cognitive diagnosis (Supplementary Table 3, Fig. 2). After correction for multiple comparisons, for males, age was the largest risk factor for WMHV followed by hypertension. For females, age was the largest risk factor for WMHV followed by hypertension, pulse pressure, diabetes, homocysteine, and HbA_1c_. All of the variables survived multiple comparisons correction for females.

**Figure 3:**
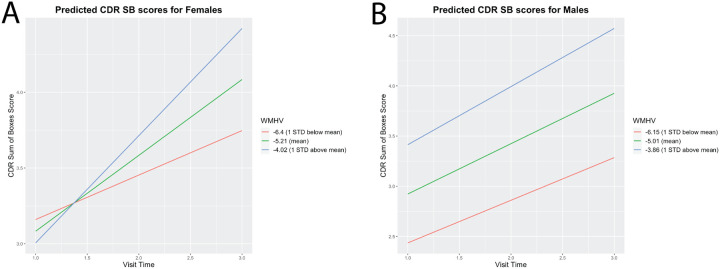
Higher WMHV is associated with clinical dementia outcomes over two years among females but not males. A.) A significant interaction effect between WMHV and visit time on CDR sum of boxes was observed for females in a linear mixed effect model. The predicted slopes for a participant with a mean and +− 1 standard deviation of WMHV for females is plotted. B.) The predicted slopes for a participant with a mean and +− 1 standard deviation of WMHV for males is plotted. There was no observed interaction effect between WMHV and visit time on CDR sum of boxes score for males. WMHV= white matter hyperintensity volume

We then explored whether baseline WMHV is differentially related to longitudinal trajectories of clinical dementia outcomes by sex (Table 4). The interaction effect between Aβ burden and visit time on CDR-SB scores was observed to be significant for both males and females. We also observed a significant interaction effect between baseline WMHV and visit time on CDR sum of boxes score for females but not males. For females, larger baseline WMHV was associated with a greater CDR sum of boxes score increase over the three visits (Fig. 3).

## Discussion/Conclusions

In a large South Korean cohort of predominantly cognitively impaired participants, we tested sex differences in WMH, in associations of known risk factors to WMH, and in associations of WMH to longitudinal clinical outcomes. Our study has three main findings. First, females appear to be protected from white matter lesions at middle age but are observed to be more vulnerable at older age compared to males. Second, diabetes status was associated with greater WMH burden in females but not males. Finally, WMHV was associated with worse clinical dementia outcomes longitudinally in females only.

A significant number of studies in the last two decades have observed elder, predominantly postmenopausal females to have a higher prevalence for cerebrovascular burden and disease compared to males^4–10, 31^. There is limited understanding of the underlying mechanisms through which these sex differences arise, but the different trajectories of endogenous sex hormones has been proposed to play a role. In particular, during the premenopause, endogenous estrogen is thought to be protective for neuronal and cerebrovascular health^32, 33^. Recently, the Rhineland study reported that postmenopausal females had more WMH compared with premenopausal females and men of the same age range^10^. Moreover, a recent UK-Biobank study observed that females with a longer reproductive lifespan had significantly smaller WMH burden in late life independent of the history of oral contraceptive use or hormone replacement therapy^34^. Taken together, our findings contribute to the growing body of literature which displays an increased risk for CSVD in late life for postmenopausal females compared to males of the same age.

Diabetes status and its severity are risk factors for CSVD. Chronic hyperglycemia stimulates the overproduction of mitochondrial superoxide radicals in endothelial cells, resulting in oxidative stress, endothelial dysfunction, and inflammation. These events are associated with the pathogenesis of vascular damage, in both small and large blood vessels^35^. Numerous cross-sectional and longitudinal studies have displayed an association between diabetes status and HbA_1c_ levels with WMH burden^18, 36–39^. Large observational studies observe type 2 diabetes confers a greater risk of incident cardiovascular disease in women compared with men^40^, and evidence also supports a more adverse effect of diabetes on CSVD in females compared to males^41^. For example, diabetes was reported to confer an increased risk of vascular dementia in females but not males in both a large community-based cohort study as well as a meta-analysis with over 2.3 million individuals^42, 43^. Some studies have also reported diabetes to be associated with greater WMH or lacune volume in females but not males^18–20^. Furthermore, animal models have supported the finding that diabetes is associated with worse cerebrovascular burden in females. A recent study observed that when mice are given a high fat diet causing hyperglycemia, white matter damage was only observed in females while neuroinflammatory activation was only observed in males^44^. Moreover, a study utilizing a genetic mouse model of diabetes reported that females had larger ischemic infarcts compared to males. These infarcts were exacerbated by ovariectomy and ameliorated by E2 treatment, which suggests estrogen directly influences diabetes effect on CSVD^45^. Collectively, our findings contribute to the idea that diabetes is differentially associated with CSVD burden between sexes. There is still limited understanding of the potential sex-specific mechanisms through which diabetes may incur sex differences in CSVD.

Marked sex differences have been reported for the trajectories of cognitive and clinical decline in dementia. In a pooled analysis of 26,000 participants, females were reported to have greater cognitive reserve but faster cognitive decline than men^46^. Data from the Alzheimer’s Disease Neuroimaging Initiative (ADNI) study displayed that female MCI participants experienced cognitive deterioration faster than males with MCI^47, 48^. Sex-specific differences in the trajectories of cognitive decline have also been supported by fMRI studies looking at memory network alterations. In particular, in a longitudinal fMRI study, we recently reported that male memory network alterations were more associated with amyloid burden while female’s alterations were more correlated to WMH volume^49^. Our longitudinal findings on clinical dementia outcomes are similar in that baseline amyloid burden was associated with worse longitudinal CDR-SB scores in both sexes, but only females are associated with worse outcomes due to baseline WMH volume. Our findings also agree with a recent longitudinal study analyzing ADNI participants, where females had worse CDR-SB scores over time compared to males with the same level of WMH burden^16^. When examining other clinical manifestations of CSVD, some studies reported that females had worse functional outcomes and cognitive decline after stroke^50, 51^. Combined, our findings suggest that elder females may have less resilience to CSVD burden compared to males.

Our study has limitations. First, we do not have information available regarding participants’ medication use. Uncontrolled hypertension has been reported to be a sex-specific risk factor for WMH^10^, thus our lack of information on participants’ medication may have contributed to our lack of findings of sex differences in associations of hypertension to WMHV. We do not have information of diabetes medication, but our findings for diabetes status can be supported by our similar observations for HbA_1c_, which is an indicator for diabetic control. Additionally, we do not have information on menopausal status nor whether participants received hormone therapy. Finally, our hospital-based cohort may have recruitment or survival bias, which has been observed to be associated with higher effect sizes for sex differences in cerebrovascular disease^3^.

In conclusion, our study provides valuable insights into the complex interplay between sex, modifiable risk factors, and clinical dementia outcomes in the context of CSVD. Elderly females appear to have an accelerated increase in cerebrovascular burden as they age, and subsequently are more vulnerable to clinical dementia decline due to CSVD. Our findings also suggest that females are more susceptible to the cerebrovascular consequences of diabetes. These findings emphasize the importance of considering sex when examining the risk factors for and cognitive sequelae of CSVD. Future research should explore the underlying mechanisms driving these sex differences and personalized prevention and treatment strategies.

## Figures and Tables

**Table 1:** Participant Characteristics

Characteristic	Group; Mean (SD)		Statistical Test	*p*-value
	Male, n = 241	Female, n = 472	t-test^a^/chi- squared test^b^	
Age	72.3 (7.44)	72.8 (7.59)	−0.93^a^	0.35
Years of Education	10.4 (4.74)	7.04 (4.54)	9.04^a^	<0.001
BMI^a^	23.9 (3.03)	24.0 (3.31)	−0.41^a^	.68
HbA_1c_^b^, % mmol/mol	6.12 (1.04)	5.98 (0.89)	−1.80^a^	0.073
log(TSH)^c^, mIU/L	0.51 (0.82)	0.46 (0.97)	−0.78^a^	0.43
log(Homocysteine)^d^, umol/L	2.69 (0.445)	2.50 (0.36)	−5.57^a^	<0.001
Global 18F-flutemetamol SUVR^e^	0.68 (0.15)	0.67 (0.15)	−0.93^a^	0.35
‡APOE ɛ4 positive, N (%)	80 (33.2%)	135 (28.6%)	1.39^b^	0.24
Hypertension, N (%)	132 (54.8%)	256 (54.2%)	0.0031^b^	0.96
Diabetes, N (%)	67 (27.8%)	93 (19.7%)	5.6^b^	0.018
Smoker, N (%)	152 (63.1%)	23 (4.9%)	288.64^b^	<0.001
**Cognitive Diagnosis, n (%)**			1.38^b^	0.50
No cognitive decline	n = 5 (2.1%)	n = 5 (1.1%)		
SCD or MCI	n = 138 (57.3%)	n = 281 (59.5%)		
Dementia	n = 98 (40.7%)	n = 186 (39.4%)		
**Cardiovascular Risk Factors**				
Pulse Pressure, mmHg	75.8 (12.4)	76.0 (11.6)	−0.24^a^	0.81
Systolic Blood Pressure, mmHg	130.7 (17.3)	134.6 (19.3)	−2.71^a^	0.0070
Diastolic Blood Pressure, mmHg	76.0 (12.1)	76.7 (11.5)	−0.70^a^	0.48
LDL-C, mg/dL	92.7 (33.3)	104 (38.8)	4.10^a^	<0.001
HDL-C, mg/dL, M (SD)	51.6 (14.1)	58.3 (14.8)	5.89^a^	<0.001

Unless otherwise indicated

a BMI information is available for all males and 471 out of 472 female participants.

b HbA_1c_ is available for 231 out of 241 male participants and 453 out of 472 female participants.

c TSH is available for all male participants and 470 out of 472 female participants.

d Homocysteine is available for 224 out of 241 male participants and 434 out of 472 femeale participants.

e Global 18F-flutemetamol SUVR is available for 209 out of 241 male participants and 435 out of 472 female participants.

† WMHV expressed as log(cm^3^/Intracranial volume)

‡ *APOE*\##\ 4 positive: 2/4, 3/4, 4/4

BMI, body mass index, DBP, diastolic blood pressure, HDL-C, high-density lipoprotein cholesterol; LDL-C, low-density lipoprotein cholesterol; SBP, systolic blood pressure, WMHV, whit matter hyperintensity volume; TSH, thyroid-stimulating hormone; SCD, subjective cognitive decline; MCI, mild cognitive impairment; AD, Alzheimer’s Disease; SUVR, standard uptake value ratio; APOE, apolipoprotein

**Table 2:** The interaction between sex with age, diabetes status is associated with WMHV. Multiple linear regression analysis tested for interaction effect with sex and known risk factors in relation to normalized white matter hyperintensity volume. Analysis controlled for scanner site and cognitive diagnosis and FDR multiple comparisons *p*-value correction was performed.

	WMHV		
	β (Std. error)	β 95% Confidence Interval	Uncorrected p-value
Age*Sex	−0.037 (0.011)	(−0.058, −0.016)	**0.00050**
Hypertension*Sex	−0.15 (0.17)	(−0.48, 0.17)	0.36
Diabetes*Sex	−0.52 (0.20)	(−0.91, −0.14)	**0.00079**
log(HbA_1c_)*Sex	−1.55 (0.63)	(−2.78, −0.32)	0.014
Pulse Pressure *Sex	−0.014 (0.0069)	(−0.028, −0.00068)	0.040
Systolic BP *Sex	−0.0062 (0.0047)	(−0.016, 0.0031)	0.19
log(Homocysteine) *Sex	−0.21 (0.21)	(−0.62, 0.21)	0.33
log(TSH) *Sex	0.17 (0.097)	(−0.025, 0.36)	0.089
Diastolic BP *Sex	−0.0076 (0.0071)	(−0.022, 0.0065)	0.29
APOE4 Status *Sex	0.27 (0.18)	(−0.089, 0.63)	0.14
HDL *Sex	0.0064 (0.0060)	(−0.0054, 0.018)	0.28
LDL*Sex	3.7E-4 (0.0024)	(−0.0043, 0.0051)	0.88
Global18F-flutemetamol SUVR *Sex	0.30 (0.60)	(−0.88, 1.47)	0.62
BMI *Sex	−0.0041 (0.027)	(−0.058, 0.050)	0.88
Smoking Status *Sex	0.10 (0.27)	(−0.42, 0.63)	0.70

Bolded font indicates the risk factor survived multiple comparisons.

BMI, body mass index; DBP, diastolic blood pressure; HDL-C, high-density lipoprotein cholesterol; LDLC, low-density lipoprotein cholesterol; SBP, systolic blood pressure; WMHV, white matter hyperintensity volume; TSH, thyroid-stimulating hormone; SUVR, standard uptake value ratio; APOE, apolipoprotein

**Table 3:** WMHV is associated with longitudinal clinical dementia outcomes in females but not males. A linear mixed effects model was utilized to test for interaction effects between WMHV*visit time, Global18Futemetamol SUVR*visit time on CDR-SB.

CDR sum of boxes			
	β (Std. error)	β 95% Confidence Interval	Uncorrected *p*-value
**Females**			
Intercept	4.69 (4.38)	(−3.93, 13.30)	0.29
Age (years)	−0.044 (0.044)	(−0.13, 0.04)	0.32
Visit Time	−0.73 (0.57)	(−1.85, 0.38)	0.20
Global18F-flutemetamol SUVR	0.34 (2.17)	(−3.94, 4.61)	0.88
WMHV	−0.18 (0.88)	(−0.75, 0.41)	0.56
Scanner Site (Site 2)	0.15 (0.87)	(−1.55, 1.86)	0.86
Scanner Site (Site 3)	−0.18 (0.88)	(−1.91, 1.55)	0.84
Scanner Site (Site 4)	0.90 (1.92)	(−2.88, 4.69)	0.64
Scanner Site (Site 5)	1.74 (2.01)	(−2.23, 5.70)	0.39
Scanner Site (Site 6)	−1.32 (0.73)	(−2.76, 0.12)	0.073
Visit Time * Global18F-flutemetamol SUVR	3.11 (0.60)	(1.92, 4.30)	**5.50e-07** ***
Visit Time * WMHV	0.18 (0.072)	(0.04, 0.32)	**0.014** *
**Males**			
Intercept	0.90 (4.60)	(−8.18, 9.98)	0.85
Age (years)	0.0084 (0.053)	(−0.10, 0.11)	0.88
Visit Time	−1.48 (-0.71)	(−2.89, −.07)	**0.039** *
Global18F-flutemetamol SUVR	2.68 (2.83)	(−2.90, 8.27)	0.34
WMHV	0.20 (0.42)	(−0.63, 1.02)	0.64
Scanner Site (Site 2)	−0.83 (1.10)	(−3.01, 1.35)	0.46
Scanner Site (Site 3)	−1.00 (1.51)	(−3.99, 1.98)	0.50
Scanner Site (Site 4)	0.39 (1.50)	(−2.58, 3.35)	0.80
Scanner Site (Site 5)	−0.30 (2.13)	(−4.51, 3.91)	0.89
**CDR sum of boxes**			
Scanner Site (Site 6)	−0.31 (1.35)	(−2.99, 2.36)	0.82
Visit Time * Global18F-flutemetamol SUVR	2.99 (0.72)	(1.56, 4.41)	**7.04e-05** ***
Visit Time * WMHV	0.019 (0.097)	(−0.17, 0.21)	0.84

* *p* < 0.05,

** *p* < 0.01,

*** *p* < 0.001

## Data Availability

The datasets generated during and/or analyzed in the current study are available from the corresponding author upon reasonable request.
